# Exon 6 of human *JAG1 *encodes a conserved structural unit

**DOI:** 10.1186/1472-6807-9-43

**Published:** 2009-07-08

**Authors:** Alessandro Pintar, Corrado Guarnaccia, Somdutta Dhir, Sándor Pongor

**Affiliations:** 1 International Centre for Genetic Engineering and Biotechnology (ICGEB), Protein Structure and Bioinformatics Group, AREA Science Park, Padriciano 99, I-34149 Trieste, Italy

## Abstract

**Background:**

Notch signaling drives developmental processes in all metazoans. The receptor binding region of the human Notch ligand Jagged-1 is made of a DSL (Delta/Serrate/Lag-2) domain and two atypical epidermal growth factor (EGF) repeats encoded by two exons, exon 5 and 6, which are out of phase with respect to the EGF domain boundaries.

**Results:**

We determined the ^1^H-NMR solution structure of the polypeptide encoded by exon 6 of *JAG1 *and spanning the C-terminal region of EGF1 and the entire EGF2. We show that this single, evolutionary conserved exon defines an autonomous structural unit that, despite the minimal structural context, closely matches the structure of the same region in the entire receptor binding module.

**Conclusion:**

In eukaryotic genomes, exon and domain boundaries usually coincide. We report a case study where this assertion does not hold, and show that the autonomously folding, structural unit is delimited by exon boundaries, rather than by predicted domain boundaries.

## Background

The Notch signaling pathway is a highly connected and tightly regulated signal transduction network that drives developmental processes in all metazoans. Notch signaling controls cell lineage decisions in tissues derived from all three primary germ lines: endoderm, mesoderm, and ectoderm thus playing an essential role in organogenesis [1-3].

**Figure 1:**
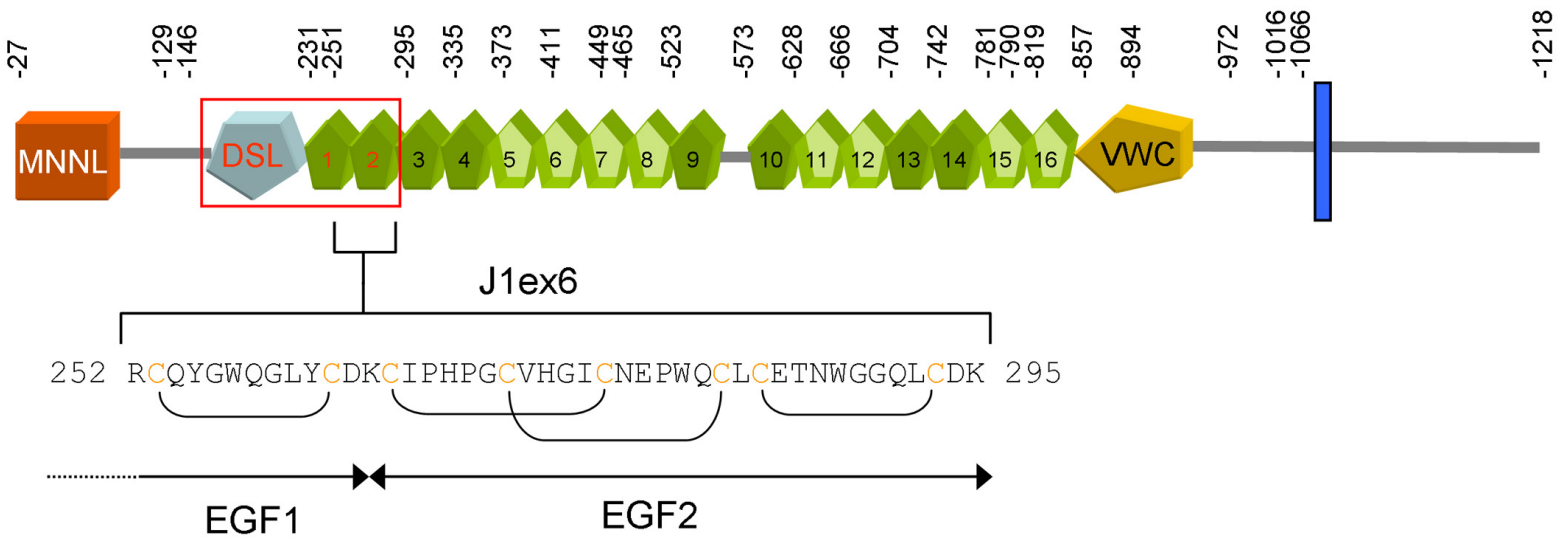
**Domain architecture of human Jagged-1**. MNNL, N-terminal domain of Notch ligands; DSL, Delta/Serrate/Lag-2 domain; EGF domains (green) are numbered progressively; potential calcium binding EGF domains are in lighter green; VWC, von Willebrand factor type C domain; the transmembrane segment is shown as a blue bar; the receptor binding region is marked in red. Amino acid number of exon boundaries are shown on top. The amino acid sequence of J1ex6 and the disulfide bond connectivities are also shown.

Both receptors and ligands are membrane-bound proteins, which normally restricts signaling to adjacent cells. Jagged-1, one of the five Notch ligands identified in man, is a single pass type I membrane protein with a large extracellular region made of a poorly characterized N-terminal region, a DSL (Delta/Serrate/Lag-2) domain, a series of 16 epidermal growth factor (EGF) tandem repeats, and a cysteine-rich juxtamembrane region (Figure 1). The DSL domain, together with the first two atypical EGF repeats constitutes Jagged-1 receptor binding region [4,5].

We previously showed [6] that a peptide corresponding to EGF2 of human Jagged-1 (residues 263–295) cannot be refolded *in vitro *in the standard oxidative folding conditions used for other EGFs. As exon 6 of the *JAG1 *gene encodes not only EGF2 but also part of EGF1, we speculated that exon 6 might encode an autonomously folding unit. We thus prepared a longer peptide encompassing the C-terminal part of EGF1 and the entire EGF2 (Figure 1). This peptide, J1ex6 (residues 252–295), could be readily refolded *in vitro *and was shown to yield a folded unit with a disulfide bond topology typical of EGF repeats [6]. We concluded that exon 6 encodes an autonomously folding unit, but whether the N-terminal overhang is only required for folding, acting as an internal chaperone in the reshuffling of disulfide bonds, or it is integral part of a structural unit encompassing the EGF1 C-terminal region and EGF2 remained an open issue.

We report here the solution structure of J1ex6 determined by ^1^H-NMR spectroscopy and demonstrate that exon 6 actually defines an EGF-like structural unit with an additional disulfide-linked loop in the N-terminal overhang. We show that the structure of this unit, in spite of the minimal structural context, is very close to the conformation of the same region in a larger construct comprising the DSL and the first three EGF repeats, for which the crystal structure has been recently determined [5]. The exon/intron organization of this region is very well conserved in this class of Notch ligands, which leads us to speculate on the evolution of this structurally peculiar and functionally relevant region.

## Results

**Figure 2:**
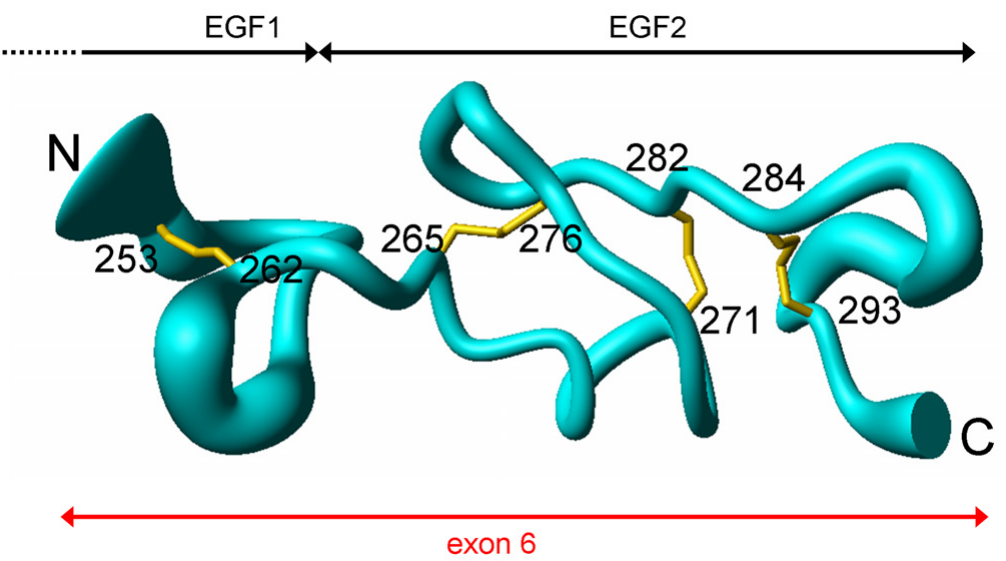
**Solution structure of J1ex6**. Backbone representation of 20 NMR models. The thickness of the trace is proportional to the backbone RMSD towards the mean. Cysteine residues are labeled with residue number and disulfide bonds are in yellow.

The solution structure of J1ex6 was determined by ^1^H NMR spectroscopy (PDB: 2KB9) (Table 1, Additional files 1 and 2). Disulfide bonds were experimentally determined by targeted proteolysis and MS analysis in a three-step strategy that lead to the unambiguous assignment of the disulfide topology, and they were explicitly used in structure calculations as distance constraints. The overall fold of J1ex6 is mainly dictated by the four disulfide bonds and lacks well defined secondary structure elements, as well as a true hydrophobic core (Figure 2). The mean pairwise RMSD values for the backbone and all heavy atoms (in parenthesis) are 1.04 ± 0.24 Å (1.65 ± 0.30 Å) from the first to the last half-cystine (residues C253–C264), 1.16 ± 0.39 Å (2.11 ± 0.52 Å) for the N-terminal overhang (residues 265–293), and 0.71 ± 0.24 Å (1.10 ± 0.26 Å) for the core EGF2 repeat (residues C265–C293). The distribution of psi/phi angles in the Ramachandran map for the 20 selected models is: 50.2% in most favored regions, 47.0% in additionally allowed regions, 2.9% in generously allowed regions, and 0.0% in disallowed regions. Whereas the availability of heteronuclear NMR data would have probably improved the precision of the models, the results for the psi/phi distribution are in line with the statistics for a set of 49 NMR structures of single and tandem EGF repeats deposited at the PDB (data not shown). It is thus possible that the sub-optimal distribution of psi/phi angles in the Ramachandran map of EGF repeats is a consequence of the constraints dictated by the disulfide bonds.

**Table 1:** Structure calculation statistics

**NMR constraints**	
Distance constraints	
Total NOE	494
Intra-residue	142
Inter-residue	352
Sequential (|*i *- *j*| = 1)	152
Medium-range (|*i *- *j*| < 5)	42
Long-range (|*i *- *j*| ≥ 5)	158
	
**Structure statistics**	
Violations*	
Upper limits (number, max value (Å))	0, 0.04
Lower limits (number, max value (Å))	0, 0.01
vdW ((number, max value (Å))	1, 0.32
Deviations from idealized geometry**	
Bond lengths, r.m.s. (Å)	0.001
Bond angles, r.m.s. (°)	0.2
Close contacts	0
Average pairwise r.m.s. deviation*** (Å)	
Heavy	2.27 ± 0.39
Backbone	1.31 ± 0.30

*Average number of violations larger than the cut-off (0.1 Å for upper and lower limits, 0.2 Å for vdW) in the 20 final structures

**From the PDB validation software

***Pairwise r.m.s. deviation calculated for the 20 final structures, all residues (252–295).

It was proposed that EGF domains can be divided in two structural groups, human EGFs (hEGF) and C1r-like EGFs (cEGF), depending on the location of the last half-cystine in the structure [7]. Using the A_N_B_N_A_C_B_C_C_N_C_C _annotation to describe the disulfide bond topology, where A_N_A_C_, B_N_B_C_, C_N_C_C _are the three disulfides, these two groups also display different lengths of the C_N_-C_C _loop, of the B_N_-A_C _loop, and of the linker connecting two EGFs of the same type. A comparison between different spacings in J1ex6 and in a set of 56 EGFs of known structure (see Additional file 3) shows that J1ex6 can be clustered together with the hEGFs for certain characteristics, such as the length of the C_N_-C_C _loop (8 residues), while for others it clusters neither with cEGFs nor with hEGFs. Notably, the B_N_-B_C _loop (10 residues) is shorter than in cEGFs (most frequently 12–13 residues) and in hEGFs (14 residues or more), as well as the total spacing between the first and the last half-cystine (A_N_-C_C _loop, 27 residues vs. 30 or more in other EGFs) and the linker between EGF1 and EGF2 (2 residues, vs. 5 or 6 in cEGFs and hEGFs, respectively). Overall, this makes J1ex6 rather more constrained than cEGFs and hEGFs. An exhaustive search of structural databases with the J1ex6 structure did not produce any hit with a significant score.

Surprisingly, the N-terminal overhang was found to be conformationally restrained and packs onto the following EGF2 unit. The interaction between the N-terminal overhang and the EGF2 repeat is mediated by a series of hydrophobic residues (Y255, W257 in EGF1; I266, P279, W280 in EGF2). This suggests that, even in solution, the EGF1-2 module is quite rigid.

**Figure 3:**
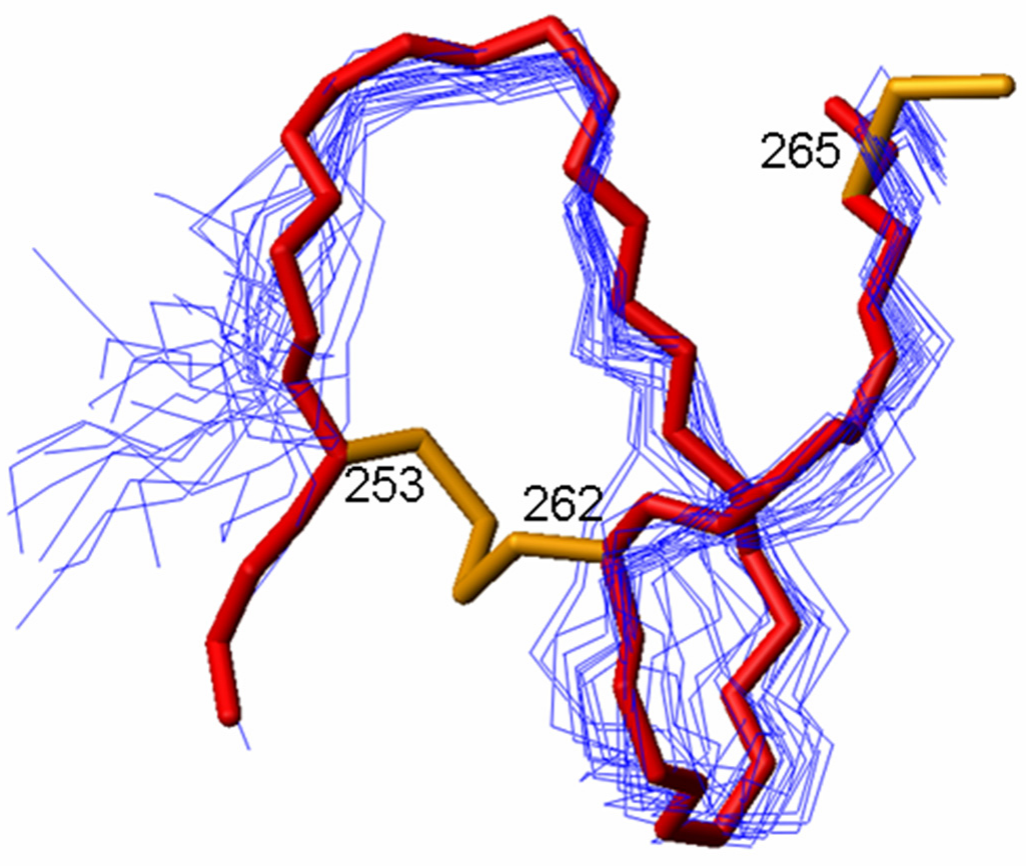
**Conformation of the N-terminal overhang**. Backbone of 20 NMR models (thin blue lines) superimposed on the backbone of the X-ray structure (thick red line) in the region 252–265, corresponding to the N-terminal overhang in J1ex6, i.e. the C-terminal loop of EGF1 in the X-ray structure. The disulfide bonds (in yellow) are shown only for the X-ray structure.

Recently, the crystal structure of the region encompassing the DSL and the first three EGF repeats of Jagged-1 has been reported [5] (PDB: 2VJ2). A comparison between the solution structure of J1ex6 and the structure of the same region in the X-ray structure shows a good agreement in the tracing of the backbone (see Additional file 4). The backbone RMSD between the X-ray structure and the 20 models of the solution structure varies between 1.97 and 2.71 Å, with an average value of 2.32 ± 0.23 Å, the largest difference being observed in the region 267–271. Interestingly, the conformation of the N-terminal overhang in the solution structure of J1ex6 is very close to that adopted by the corresponding segment in the crystal structure of the DSL/EGF1-3 tandem domains, with an average RMSD of 1.86 ± 0.24 Å for the region 253–265 (Figure 3). These results confirm that exon 6 of the human *JAG1 *gene is actually encoding a structural unit containing three disulfide bonds with an EGF-like topology and an additional disulfide bond that is N-terminal to the EGF domain, rather than C-terminal as in laminin EGF-like domains.

**Figure 4:**
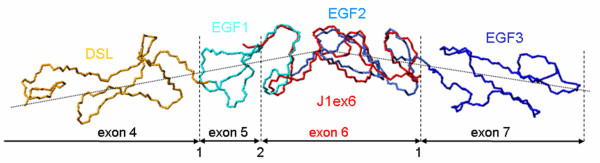
**Structure of the receptor binding region**. The X-ray model the DSL domain and the first three EGFs of Jagged-1 (PDB: 2VJ2) superimposed on the solution structure of J1ex6 (PDB: 2KB9, first model, in red); exon boundaries and phases are also shown.

Furthermore, in the crystal structure of the DSL/EGF1-3 modules [5], a kink is present between EGF1 and EGF2 in an otherwise linear, rod-like structure (Figure 4). Because this construct crystallized as a dimer with several interchain contacts, it can be questioned if packing forces are responsible for the bending of the chain. On the other hand, the good agreement between the crystal structure and the solution structure, in particular in the N-terminal overhang, and despite the reduced structural context, suggests that the kink is actually a structural feature that might have some functional relevance.

To find out if the dephasing of exon boundaries with respect to predicted domain boundaries in the region comprising these two atypical EGF repeats is accidental, or might underlie some common evolutionary origin, we analyzed the exon/intron organization of human *JAG1 *orthologues in 26 different species including primates (5), non-primate mammals (15), birds (1), amphibians (1), and fishes (4). The exon/intron arrangement in this region of the *JAG1 *genes is very well conserved throughout evolution, with a single exon encoding the C-terminal region of EGF1 and the complete EGF2 (see Additional file 5). The extension of this analysis to all homologues of Notch ligands showed that the same exonic organization is found not only in *JAG1 *but also in the *JAG2*, *DLL1*, *DLL4*, *DLK1*, and *DLK2 *gene families, for a total of 112 genes in species varying from fishes to primates, and only three exceptions found, all in lower organisms (see Additional files 6 and 7). Usually, exon 6 (or its equivalent) is flanked by a phase 2 and a phase 1 intron on the 5' and 3' ends, respectively.

## Discussion

Early on in 1978 it was proposed that exons encode "folded protein units", emphasizing the role of a correct folding process to produce functional proteins or domains [8]. Recent advances in genome sequencing, domain classification, and 3D structure determination confirmed this hypothesis: a strong correlation between exon boundaries and predicted domain boundaries has been found in nine eukaryotic genomes, the correlation becoming stronger as the genome complexity becomes higher [9]. Such a high correlation lead to the suggestion that in certain cases exon boundaries can be used to predict domain limits more accurately [10]. In particular, a survey of domain repeats in seven metazoan species showed that there is a very good correspondence between exons and EGF repeats (0.93 exon/repeat on the average) [11]. In the case reported here, exon boundaries do not coincide with the expected EGF domain limits. Although it can be argued that in some instances domain limits cannot be defined precisely, this is not the case of EGF repeats, which are clearly recognizable by a very specific pattern of the three disulfide bonds and by the spacing between half-cystines. In this case study, the overall correspondence is maintained, with exons 5 and 6 encoding EGF1 and 2, but exon and domain boundaries are clearly out of phase, with exon 5 encoding a truncated EGF with only four half-cystines and exon 6 encoding the C-terminal half of EGF1 and the entire EGF2. Furthermore, this peculiar exon/intron organization seems to be well conserved throughout evolution. How can these results be reconciled with the experimental finding that exon 6 of human *JAG1 *is encoding an autonomously folding and structural unit? Although from the statistical point of view this may be one of the rare instances where the 1:1 correspondence between exons and EGF repeat does not hold, the question remains if this has any structural or functional significance. It is possible that the particular exon structure in this region is dictated by folding and structural requirements. In this specific case, the constraints in the atypically short EGF2 repeat might require the N-terminal extension as an internal chaperone and a docking template to drive the correct folding.

Furthermore, the interface between EGF1 and EGF2 drives the relative orientation of the EGF1-2 tandem repeats and may have a functional role. It was shown that deletion of the DSL domain in a Jagged-1 construct abolishes binding to Notch [4]. Whereas the DSL domain is necessary for binding, it is not sufficient. A construct containing the MNNL region and the DSL domain binds only weakly, while addition of the EGF1-2 restores full binding [4]. Although the structural determinants of the interaction between DSL ligands and Notch receptors are not known in detail yet, the presence of a kink at the interface between EGF1 and EGF2 observed in the crystal structure of the Jagged-1 region comprising the DSL domain and the first three EGF repeats [5] might not be accidental and may be required for correct binding to Notch receptors. In calcium binding EGFs, which are connected by a fairly long linker, the relative orientation of two adjacent domains is mainly determined by the geometric constraints imposed by the coordination of the calcium ion. In EGF1-2, the same objective is achieved by drastically reducing the length of the linker region and encoding the C-terminal part of EGF1 and EGF2 in a single, conserved exon.

It has been proposed that the DSL domain may have evolved from the truncation of tandemly connected, short EGF domains [5]. In fact, J1ex6 in itself can be viewed as two truncated tandem EGFs, and the sequence and disulfide pattern similarities between the DSL domain and J1ex6 are actually significant (see Additional file 8). One might then ask whether there is any evolutionary relationship between the two or, in other words, if the DSL domain and J1ex6 might have arisen from duplication of a common ancestor followed by divergent evolution and loss of one disulfide linkage in the DSL domain. If this hypothesis is true, one should be able to identify a primitive precursor where either the DSL or J1ex6 is missing. Indeed, we identified the non-canonical Notch ligands DLK1 and DLK2 as hits sharing with *JAG1 *a high sequence similarity and the same exon organization in the region comprising EGF1 and 2. Interestingly, these proteins lack the DSL domain, and this makes them good candidates as precursors of canonical Notch ligands. However, DLK1 and 2 are found only in vertebrates, and not in more primitive organisms such as nematodes and insects. [Note added in proof: After acceptance of our manuscript, Dr. Anne C. Hart called our attention to a paper recently published by her group in PLOS Biology (6(8):196, 2008) in which it is proposed that the secreted *C. elegans* protein OSM-11 is a functional ortholog of mammalian DLK1]. Furthermore, the DSL domain is made not only of a cysteine-rich region, but also of a more variable N-terminal region that is usually encoded by the same exon. The genome of the microbal eukaryote *Monosiga brevicollis*, one of the closest primitive relatives of metazoans, has been recently sequenced and revealed some archetypal features of Notch signaling [12]. Domains that are typical of Notch receptor proteins, such as Notch/Lin, ankyrin, and EGF repeats are already present, although in distinct proteins and not arranged in the same domain architecture as in metazoan Notch proteins, but not homologues of Notch ligands. We were not able as well to detect any homologue of the DSL domain in the genome of *M. brevicollis*, but we found several hits corresponding to short EGF repeats. In conclusion, currently available data still do not provide strong evidence of an evolutionary relationship between the DSL domain and J1ex6, but are in support of a later appearance of the DSL domain with respect to the short EGF repeats. The unusual exon architecture of the region comprising the EGF1 and EGF2 repeats might have arisen from the insertion of an intron in a common precursor encoding both EGF1 and EGF2, and then conserved during the evolution of metazoans, together with the amino acid sequence.

## Conclusion

In eukaryotic genomes, there is an overall very good correspondence between exon boundaries and predicted domain limits [9-11]. We report a case study where this correspondence is not fulfilled, and show that the autonomously folding, structural unit is defined by exon boundaries, rather than by predicted domain boundaries. Although this conclusion cannot be taken as a general rule, this study suggests that, together with domain boundaries and predicted secondary structure, exon boundaries may also be taken into account when designing constructs for structural studies. This option should be carefully considered especially when dealing with protein regions for which no similarity with known domains can be detected. These regions, also called "orphan domains", account for as much as ~15% of the eukaryotic proteomes [13], while an additional ~30% is made of poorly characterized regions such as those belonging to the Pfam-B families [14].

## Methods

### Peptide synthesis

J1ex6 (44 amino acid long, corresponding to residues 252–295 of human Jagged-1) was synthesized on solid phase using Fmoc/tBu chemistry as previously described [6]. Cysteine residues were introduced by double coupling as N-α-Fmoc-S-trityl-L-cysteine pentafluorophenyl ester in order to avoid cysteine racemization. All other amino acids were introduced as double couplings using a 4× excess of amino acid (Fmoc-AA/HCTU/DIPEA = 1/1/2). After cleavage/deprotection, the peptide was precipitated with diethylether, washed and freeze-dried. The crude peptide was reduced by TCEP and purified by RP-HPLC on a Zorbax 300SB-C18 semipreparative column. The purified peptide fractions were diluted to a final peptide concentration of 0.1 mg/mL in the degassed refolding buffer (0.25 M Tris-HCl, 2 mM EDTA, 3.7 mM GSH, 3.7 mM GSSG, pH 8) and refolded for 18 hours at 4°C. After acid quenching of the folding reaction with TFA, J1ex6 was purified by RP-HPLC using a Zorbax SB300-C18 column and freeze-dried.

The complete disulfide pattern of the folded peptide was unambiguously determined by targeted proteolysis and MS analysis in three steps. In the first reaction, the purified peptide (160 μg) was dissolved in 250 μL of sodium acetate buffer (50 mM, pH 5.6) containing 5 mM CaCl_2 _and incubated with trypsin (8 μg) for 18–48 h at 37°C. The reaction mixture was further incubated for 48 h at 37°C in the presence of thermolysin (15 μg). A fragment corresponding to the two-disulfide-bonded core was then isolated by RP-HPLC and subjected to a further proteolysis with proline-endopeptidase (1/20 w/w) for 18 h. At each step, aliquots from the digestion mixtures were desalted by ZipTip C18 (Millipore), mixed (1:1) with MALDI matrix (10 mg/mL HCCA in 75% MeCN/25% H_2_O/0.1% TFA) and analyzed by MALDI-MS on an Applied Biosystems 4800 TOF/TOF Analyzer operated in reflectron positive ion mode.

### NMR

The sample for NMR spectroscopy was prepared dissolving the freeze-dried peptide in H_2_O/D_2_O (90/10, v/v) for a final sample concentration of ~0.5 mM and adjusting the pH to ~4.5 with NaOH 0.1 N. Limited solubility hampered data acquisition at higher pH values. Spectra were recorded at 298 K on a Bruker Avance operating at a ^1^H frequency of 800.13 MHz and equipped with a triple resonance, z-axis gradient cryo-probe. 2D NOESY and TOCSY spectra were recorded using 150 ms and 80 ms mixing times, respectively. Additional spectra were recorded on the same sample dissolved in D_2_O. Data were transformed using X-WinNMR (Bruker) and analyzed using CARA [15]. Chemical shifts were referenced to internal DSS. Assignment of ^1^H backbone and side-chain resonances was achieved from COSY, TOCSY, and NOESY spectra using standard techniques. Structure calculations were carried out in a completely automated fashion using CYANA 2.1 [16]. Disulfide bonds were explicitly added as distance constraints, with the weight for the upper SG-SG distance set to 10. Distance constraints were derived starting from 922 peaks manually picked in NOESY spectra recorded in H_2_O/D_2_O (90/10, v/v) and in D_2_O, and automatically assigned in a recursive manner within the standard CYANA protocol using 0.030 and 0.040 ppm chemical shift tolerance in the detected and indirect ^1^H dimensions, respectively. In each calculation round, 100 structures were minimized and 20 models were finally selected according to the target function value. Coordinates were deposited at the PDB (PDB code: 2KB9). Figures were prepared using MOLMOL [17].

### Sequence analysis

Conservation of exon boundaries in 26 orthologues of human *JAG1 *retrieved from ENSEMBLE was verified by a BLAST search of the human J1ex6 amino acid sequence over the entire set of translated exons. The same type of search was extended to all homologues of human Jagged-1 for a total of 112 sequences. Sequences were then aligned using CLUSTAL-W.

## Authors' contributions

AP conceived the study, determined the NMR structure, and wrote the manuscript. CG carried out the peptide synthesis, purification, and disulfide bond determination. SD performed the sequence analysis. SP coordinated the study, participated in its design and contributed to draft the manuscript. All authors read and approved the final manuscript.

## Supplementary Material

Additional file 1 **NMR structure determination**. Statistics from CYANA structure calculation cycles. Click here for file

Additional file 2 **NMR structure**. Stereo-view of J1ex6 (20 models); backbone in blue, side chains in cyan, disulfide bonds in orange; the first and last residues are not shown. Click here for file

Additional file 3 **Classification of EGF repeats**. Different spacings calculated for a dataset of 56 structures classified as cEGF (empty bars) or hEGF (filled bars); spacings in EGF2 are marked by an asterisk. Click here for file

Additional file 4 **Structure comparison**. Overlay of the J1ex6 solution structure (20 models, blue lines) with the backbone of the same region in the crystal structure (PDB: 2VJ2; left, chain A; right, chain B). Click here for file

Additional file 5 **Exon/intron organization**. Diagrams showing exon/intron organization, intron phase, and domain architecture in the DSL/EGF1-3 region of human Jagged-1 (JAG1_HUMAN). The same exon/intron organization, with a single exon encoding the C-terminal region of EGF1 and the entire EGF2, is shared by all the 112 homologues of human Jagged-1 used in the multiple sequence alignment. Outliers displaying a different exon/intron organization are also shown. In *Drosophila *Delta (DL_DROME) exon 6 is encoding not only the C-terminal region of EGF1 and the entire EGF2 but also the following EGFs; in *C. elegans *APX1 (APX1_CAEEL) a single exon is encoding both EGF1 and EGF2; in zebrafish Delta-like B (DLLB_DANRE) a single exon is encoding EGFs1-3. To identify these outliers, Swiss-Prot was searched for all proteins containing EGF repeats, entries for which the exon/intron organization is annotated in ENSEMBLE were collected, amino acid sequences broken down into segments corresponding to exons, and a BLAST search was performed with the sequence encoded by Jagged-1 exon 6. Click here for file

Additional file 6 **Sequence analysis**. List of genes used for the multiple sequence alignment of the polypeptides encoded by exon 6 of human *JAG1*. Click here for file

Additional file 7 **Sequence alignment**. Multiple sequence alignment of the polypeptides encoded by exon 6 of human *JAG1 *and its homologues in different species. All amino acid sequences annotated in ENSEMBLE as orthologues to *JAG1, JAG2, DLL1, DLL4, DLK1, and DLK2 *were collected, broken down into segments corresponding to exons, and searched using BLAST with the sequence encoded by exon 6 of human Jagged-1; hits were then aligned using CLUSTAL-W. Click here for file

Additional file 8 **Sequence comparison**. Multiple sequence alignment of the amino acid sequence encoded by exon 6 in *JAG1 *and its homologues compared to the sequence of the DSL domain; the disulfide topology in the DSL and J1ex6 region of human Jagged-1 is also shown. Click here for file
