# Supplementary material for: Exon 6 of human JAG1 encodes a conserved structural unit
Source: BMC Struct Biol. 2009 Jul 8;9:43. doi: 10.1186/1472-6807-9-43 (PMC2725086; doi:10.1186/1472-6807-9-43)
Supplement: Additional file 1 — NMR structure determination. Statistics from CYANA structure calculation cycles. [file 1472-6807-9-43-S1.pdf]

Cycle	1	2	3	4	5	6	7	final
Peaks:								
selected	922	922	922	922	922	922	922	
assigned	759	812	807	811	807	806	804	
unassigned	163	110	115	111	115	116	118	
Cross peaks:								
off-diagonal	759	812	807	811	807	806	804	
unique	261	586	622	635	662	680	680	
short-range |i-j|<=1	584	606	600	597	595	588	587	
medium-range 1<|i-j|<5	54	38	41	44	39	41	36	
long-range |i-j|>=5	121	168	166	170	173	177	181	
Upper limits:								
total	475	474	466	464	457	458	486	494
short-range |i-j|<=1	323	315	308	300	296	292	291	294
medium-range 1<|i-j|<5	83	61	39	41	36	38	40	42
long-range |i-j|>=5	69	98	119	123	125	128	155	158
Average assign./constr.	4.10	1.83	1.29	1.26	1.20	1.17	1.00	1.00
Average f value	9.10	4.90	4.44	0.87	0.63	0.46	0.41	0.38
Average RMSD to mean								
backbone	4.36	1.76	0.84	0.93	0.87	1.00	0.90	0.90
heavy atoms	5.23	2.53	1.47	1.62	1.50	1.60	1.50	1.57
