# Supplementary figures and images for: Exon 6 of human JAG1 encodes a conserved structural unit
Source: BMC Struct Biol. 2009 Jul 8;9:43. doi: 10.1186/1472-6807-9-43 (PMC2725086; doi:10.1186/1472-6807-9-43)

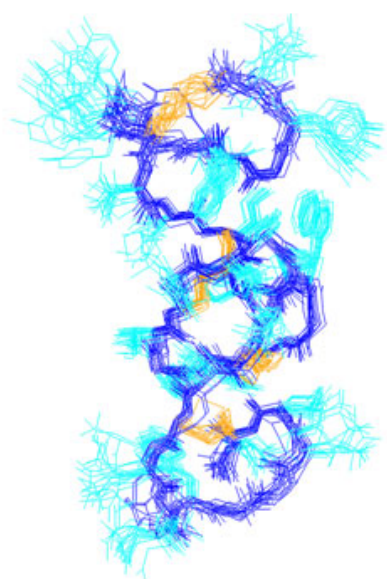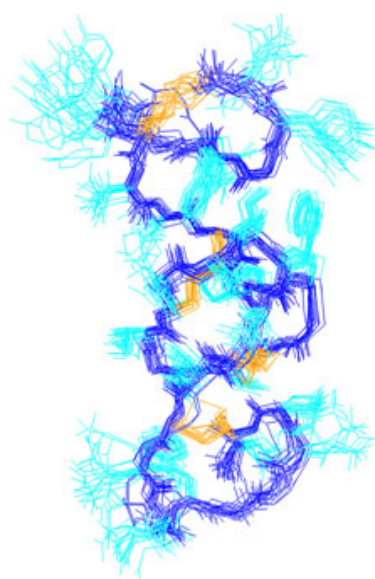

Supplement: Additional file 2 — NMR structure. Stereo-view of J1ex6 (20 models); backbone in blue, side chains in cyan, disulfide bonds in orange; the first and last residues are not shown. [file 1472-6807-9-43-S2.pdf]

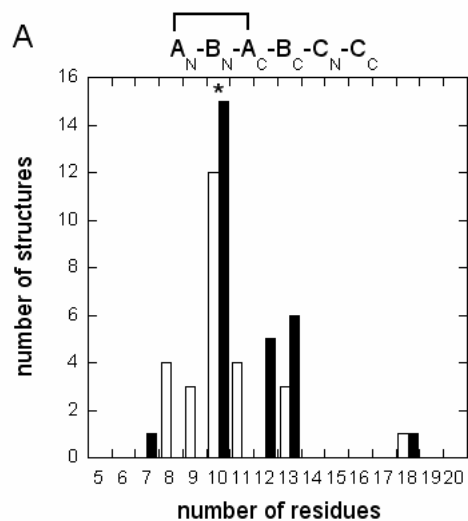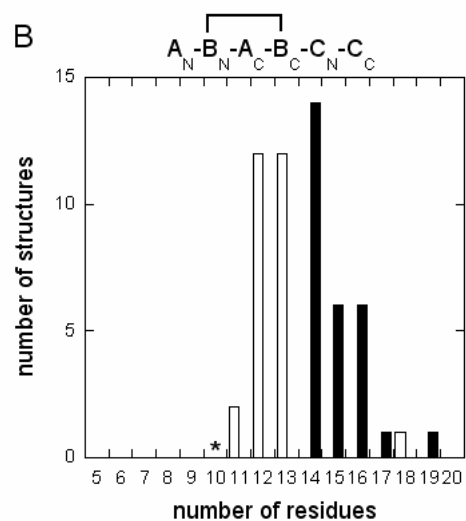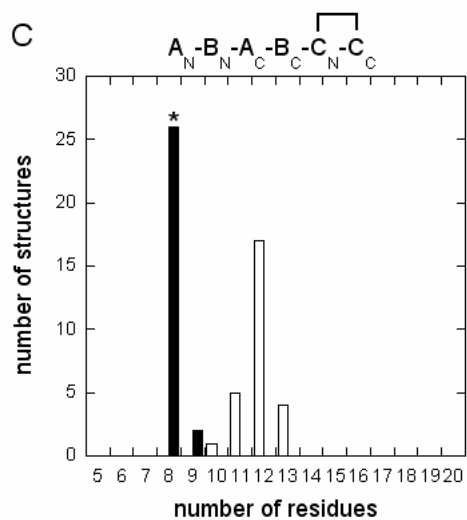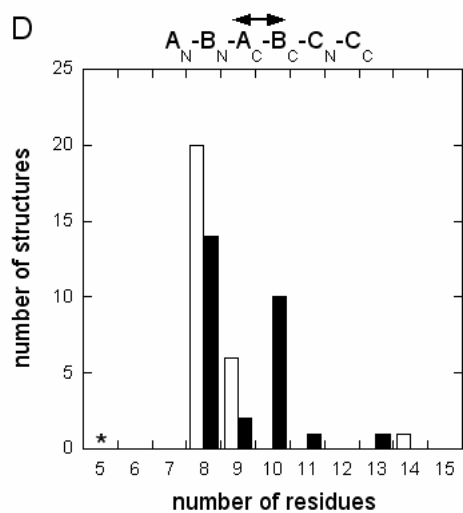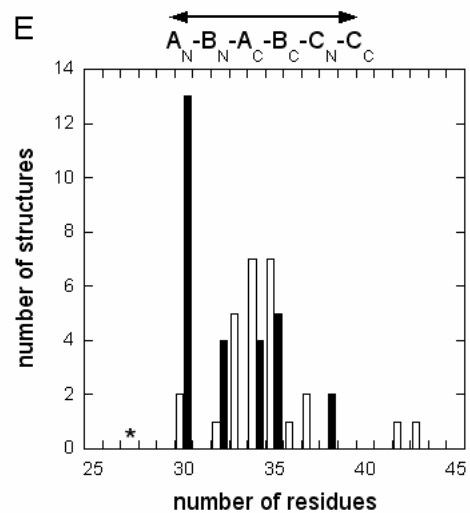

Supplement: Additional file 3 — Classification of EGF repeats. Different spacings calculated for a dataset of 56 structures classified as cEGF (empty bars) or hEGF (filled bars); spacings in EGF2 are marked by an asterisk. [file 1472-6807-9-43-S3.pdf]

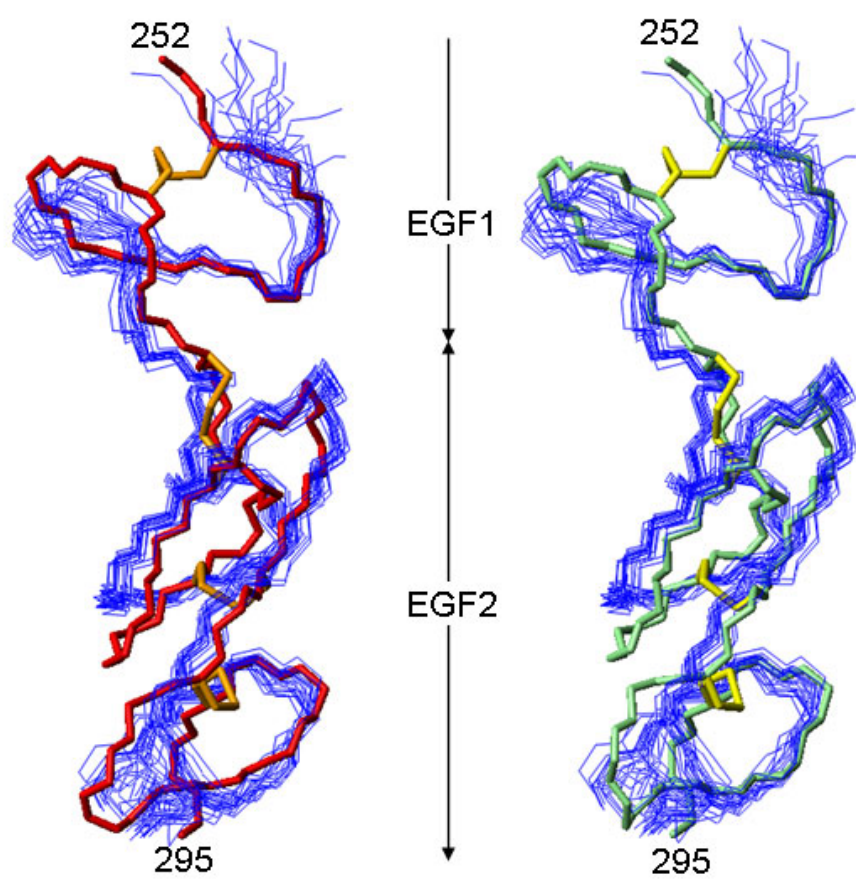

Supplement: Additional file 4 — Structure comparison. Overlay of the J1ex6 solution structure (20 models, blue lines) with the backbone of the same region in the crystal structure (PDB: 2VJ2; left, chain A; right, chain B). [file 1472-6807-9-43-S4.pdf]

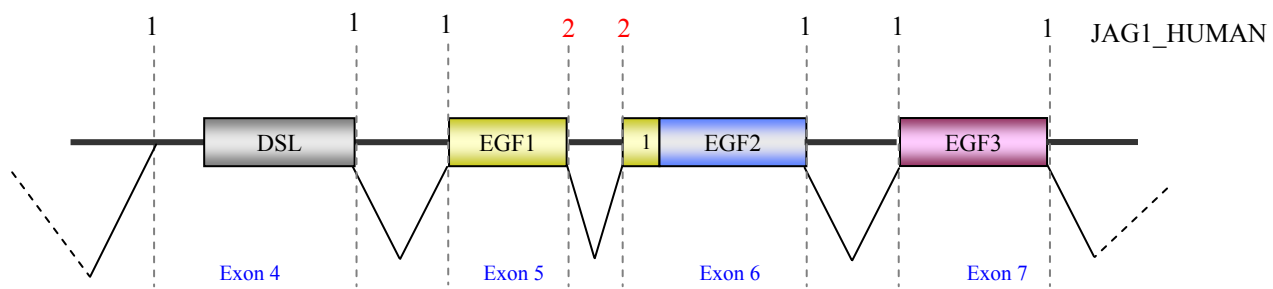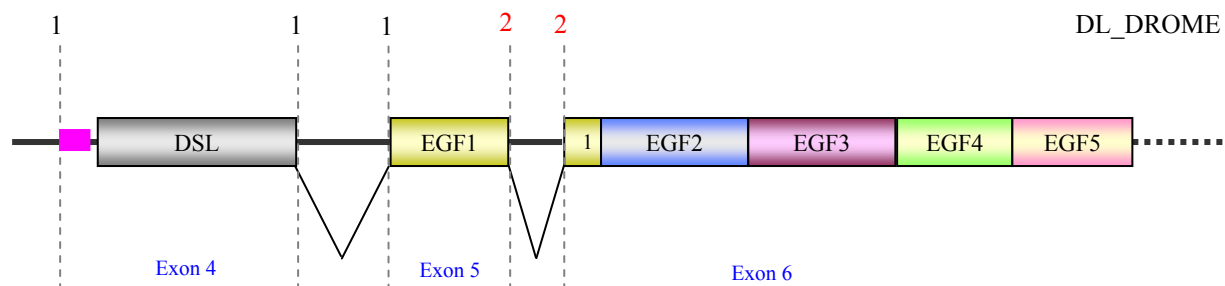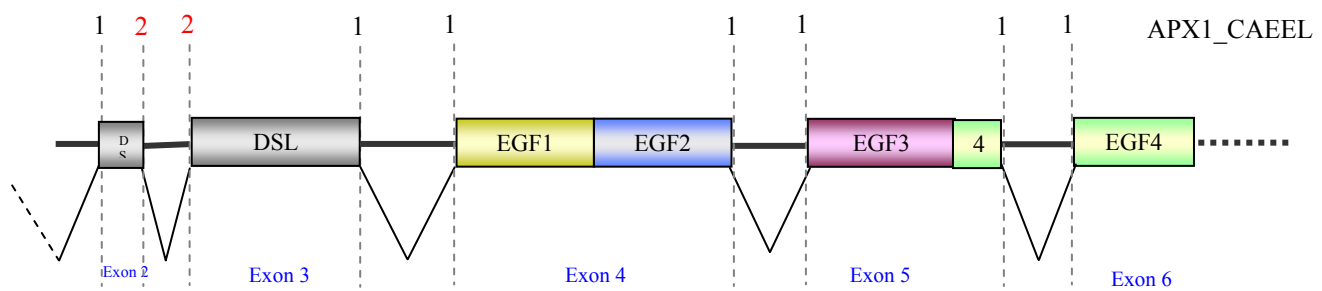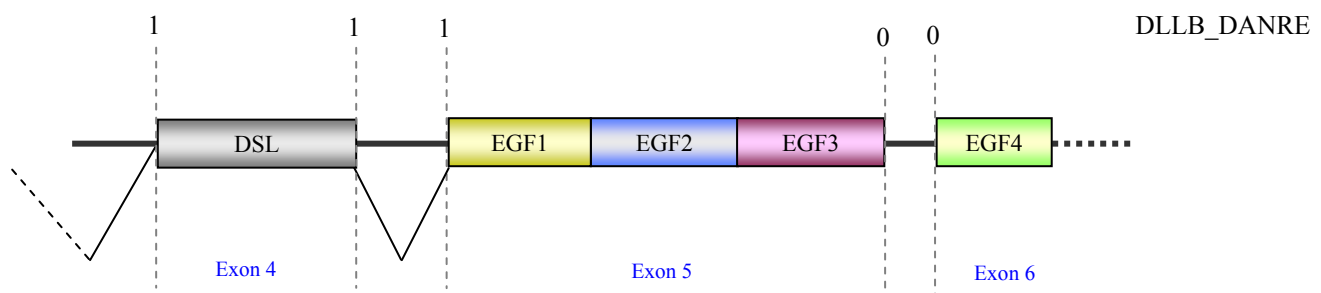

Supplement: Additional file 5 — Exon/intron organization. Diagrams showing exon/intron organization, intron phase, and domain architecture in the DSL/EGF1-3 region of human Jagged-1 (JAG1_HUMAN). The same exon/intron organization, with a single exon encoding the C-terminal region of EGF1 and the entire EGF2, is shared by all the 112 homologues of human Jagged-1 used in the multiple sequence alignment. Outliers displaying a different exon/intron organization are also shown. In Drosophila Delta (DL_DROME) exon 6 is encoding not only the C-terminal region of EGF1 and the entire EGF2 but also the following EGFs; in C. elegans APX1 (APX1_CAEEL) a single exon is encoding both EGF1 and EGF2; in zebrafish Delta-like B (DLLB_DANRE) a single exon is encoding EGFs1-3. To identify these outliers, Swiss-Prot was searched for all proteins containing EGF repeats, entries for which the exon/intron organization is annotated in ENSEMBLE were collected, amino acid sequences broken down into segments corresponding to exons, and a BLAST search was performed with the sequence encoded by Jagged-1 exon 6. [file 1472-6807-9-43-S5.pdf]
