# Supplementary material for: Exon 6 of human JAG1 encodes a conserved structural unit
Source: BMC Struct Biol. 2009 Jul 8;9:43. doi: 10.1186/1472-6807-9-43 (PMC2725086; doi:10.1186/1472-6807-9-43)
Supplement: Additional file 6 — Sequence analysis. List of genes used for the multiple sequence alignment of the polypeptides encoded by exon 6 of human JAG1. [file 1472-6807-9-43-S6.doc]

| **Gene** | **Exon No** | **Species** | **Ensemble ID** | **From** | **To** |
| --- | --- | --- | --- | --- | --- |
| JAG1 | 6 | Homo sapiens | ENSG00000101384 | 255 | 299 |
| JAG1 | 6 | Macaca mulatta | ENSMMUG00000018116 | 255 | 299 |
| JAG1 | 6 | Pan troglodytes | ENSPTRG00000013250 | 255 | 299 |
| JAG1 | 6 | Pongo pygmaeus | ENSPPYG00000010708 | 255 | 299 |
| JAG1 | 7 | Microcebus murinus | ENSMICG00000005608 | 255 | 299 |
| JAG1 | 6 | Rattus norvegicus | ENSRNOG00000007443 | 255 | 299 |
| JAG1 | 6 | Mus musculus | ENSMUSG00000027276 | 255 | 299 |
| JAG1 | 10 | Ochotona princeps | ENSOPRG00000008390 | 229 | 273 |
| JAG1 | 8 | Oryctolagus cuniculus | ENSOCUG00000002884 | 254 | 298 |
| JAG1 | 6 | Bos taurus | ENSBTAG00000012817 | 255 | 299 |
| JAG1 | 5 | Equus caballus | ENSECAG00000012993 | 229 | 273 |
| JAG1 | 7 | Loxodonta africana | ENSLAFG00000018468 | 256 | 300 |
| JAG1 | 6 | Canis familiaris | ENSCAFG00000005627 | 255 | 299 |
| JAG1 | 6 | Felis catus | ENSFCAG00000002195 | 255 | 299 |
| JAG1 | 5 | Myotis lucifugus | ENSMLUG00000009421 | 228 | 272 |
| JAG1 | 14 | Sorex araneus | ENSSARG00000005039 | 252 | 296 |
| JAG1 | 6 | Dasypus novemcinctus | ENSDNOG00000007593 | 255 | 299 |
| JAG1 | 6 | Monodelphis domestica | ENSMODG00000004910 | 254 | 298 |
| JAG1 | 6 | Echinops telfairi | ENSETEG00000001437 | 255 | 299 |
| JAG1 | 6 | Ornithorhynchus anatinus | ENSOANG00000008425 | 254 | 298 |
| JAG1 | 5 | Gallus gallus | ENSGALG00000009020 | 229 | 273 |
| JAG1 | 6 | Xenopus tropicalis | ENSXETG00000002340 | 255 | 299 |
| JAG1 | 6 | Oryzias latipes | ENSORLG00000000972 | 256 | 300 |
| JAG1 | 6 | Tetraodon nigroviridis | ENSTNIG00000016644 | 254 | 298 |
| JAG1 | 4 | Danio rerio | ENSDARG00000030289 | 129 | 173 |
| JAG1 | 5 | Gasterosteus aculeatus | ENSGACG00000004493 | 233 | 277 |
| JAG2 | 6 | Homo sapiens | ENSG00000184916 | 266 | 310 |
| JAG2 | 4 | Macaca mulatta | ENSMMUG00000001276 | 127 | 171 |
| JAG2 | 4 | Pongo pygmaeus | ENSPPYG00000006203 | 127 | 170 |
| JAG2 | 6 | Mus musculus | ENSMUSG00000002799 | 264 | 308 |
| JAG2 | 5 | Rattus norvegicus | ENSRNOG00000013927 | 220 | 264 |
| JAG2 | 4 | Cavia porcellus | ENSCPOG00000008419 | 127 | 171 |
| JAG2 | 4 | Equus caballus | ENSECAG00000006609 | 129 | 173 |
| JAG2 | 1 | Bos taurus | ENSBTAG00000007319 | 1 | 45 |
| JAG2 | 4 | Canis familiaris | ENSCAFG00000018401 | 127 | 171 |
| JAG2 | 3 | Monodelphis domestica | ENSMODG00000014707 | 107 | 151 |
| JAG2 | 3 | Ornithorhynchus anatinus | ENSOANG00000007869 | 104 | 148 |
| JAG2 | 5 | Gallus gallus | ENSGALG00000011696 | 235 | 279 |
| JAG2 | 6 | Gasterosteus aculeatus | ENSGACG00000007522 | 259 | 303 |
| JAG2 | 7 | Takifugu rubripes | ENSTRUG00000000042 | 263 | 307 |
| JAG2 | 1 | Tetraodon nigroviridis | ENSTNIG00000012383 | 1 | 45 |
| JAG2 | 6 | Oryzias latipes | ENSORLG00000017877 | 259 | 303 |
| JAG2 | 6 | Danio rerio | ENSDARG00000021389 | 258 | 302 |
| DLL1 | 6 | Homo sapiens | ENSG00000198719 | 247 | 291 |
| DLL1 | 5 | Macaca mulatta | ENSMMUG00000021144 | 197 | 241 |
| DLL1 | 6 | Pongo pygmaeus | ENSPPYG00000017189 | 247 | 291 |
| DLL1 | 6 | Pan troglodytes | ENSPTRG00000018824 | 310 | 354 |
| DLL1 | 6 | Rattus norvegicus | ENSRNOG00000014667 | 246 | 290 |
| DLL1 | 6 | Mus musculus | ENSMUSG00000014773 | 246 | 290 |
| DLL1 | 6 | Oryctolagus cuniculus | ENSOCUG00000013290 | 246 | 290 |
| DLL1 | 6 | Bos taurus | ENSBTAG00000031476 | 247 | 291 |
| DLL1 | 6 | Canis familiaris | ENSCAFG00000004094 | 197 | 241 |
| DLL1 | 3 | Felis catus | ENSFCAG00000004661 | 108 | 152 |
| DLL1 | 7 | Sorex araneus | ENSSARG00000007078 | 126 | 170 |
| DLL1 | 6 | Monodelphis domestica | ENSMODG00000005607 | 259 | 303 |
| DLL1 | 2 | Erinaceus europaeus | ENSEEUG00000007260 | 21 | 65 |
| DLL1 | 4 | Myotis lucifugus | ENSMLUG00000005071 | 130 | 174 |
| DLL1 | 6 | Gallus gallus | ENSGALG00000011182 | 254 | 298 |
| DLL1 | 6 | Xenopus tropicalis | ENSXETG00000022525 | 249 | 293 |
| DLL1 | 5 | Tupaia belangeri | ENSTBEG00000014817 | 131 | 175 |
| DLL1 | 6 | Takifugu rubripes | ENSTRUG00000006183 | 249 | 293 |
| DLL1 | 6 | Oryzias latipes | ENSORLG00000010606 | 249 | 293 |
| DLL1 | 6 | Gasterosteus aculeatus | ENSGACG00000016131 | 250 | 294 |
| DLL4 | 6 | Homo sapiens | ENSG00000128917 | 317 | 361 |
| DLL4 | 7 | Pan troglodytes | ENSPTRG00000006937 | 316 | 360 |
| DLL4 | 6 | Macaca mulatta | ENSMMUG00000014541 | 243 | 287 |
| DLL4 | 7 | Otolemur garnettii | ENSOGAG00000001215 | 244 | 288 |
| DLL4 | 6 | Microcebus murinus | ENSMICG00000005797 | 243 | 287 |
| DLL4 | 6 | Rattus norvegicus | ENSRNOG00000014011 | 244 | 288 |
| DLL4 | 6 | Mus musculus | ENSMUSG00000027314 | 244 | 288 |
| DLL4 | 4 | Cavia porcellus | ENSCPOG00000011383 | 111 | 155 |
| DLL4 | 11 | Ochotona princeps | ENSOPRG00000000813 | 318 | 362 |
| DLL4 | 7 | Oryctolagus cuniculus | ENSOCUG00000010756 | 243 | 287 |
| DLL4 | 6 | Equus caballus | ENSECAG00000013434 | 243 | 287 |
| DLL4 | 6 | Bos taurus | ENSBTAG00000010361 | 243 | 287 |
| DLL4 | 6 | Canis familiaris | ENSCAFG00000009401 | 243 | 287 |
| DLL4 | 9 | Felis catus | ENSFCAG00000014721 | 316 | 360 |
| DLL4 | 3 | Myotis lucifugus | ENSMLUG00000004545 | 111 | 155 |
| DLL4 | 6 | Sorex araneus | ENSSARG00000005952 | 244 | 288 |
| DLL4 | 6 | Monodelphis domestica | ENSMODG00000000198 | 244 | 288 |
| DLL4 | 3 | Erinaceus europaeus | ENSEEUG00000014146 | 110 | 154 |
| DLL4 | 16 | Tupaia belangeri | ENSTBEG00000010805 | 240 | 284 |
| DLL4 | 3 | Ornithorhynchus anatinus | ENSOANG00000012601 | 61 | 105 |
| DLL4 | 6 | Gallus gallus | ENSGALG00000008514 | 243 | 287 |
| DLL4 | 5 | Xenopus tropicalis | ENSXETG00000021584 | 231 | 275 |
| DLL4 | 5 | Gasterosteus aculeatus | ENSGACG00000005896 | 218 | 262 |
| DLL4 | 6 | Danio rerio | ENSDARG00000070425 | 236 | 280 |
| DLL4 | 8 | Oryzias latipes | ENSORLG00000016743 | 250 | 294 |
| DLL4 | 8 | Takifugu rubripes | ENSTRUG00000012962 | 236 | 280 |
| DLL4 | 6 | Tetraodon nigroviridis | ENSTNIG00000010969 | 233 | 277 |
| DLK1 | 3 | Homo sapiens | ENSG00000185559 | 46 | 90 |
| DLK1 | 3 | Macaca mulatta | ENSMUSG00000040856 | 46 | 90 |
| DLK1 | 3 | Pongo pygmaeus | ENSPPYG00000006146 | 46 | 90 |
| DLK1 | 1 | Otolemur garnettii | ENSOGAG00000004875 | 1 | 44 |
| DLK1 | 3 | Rattus norvegicus | ENSRNOG00000019584 | 46 | 90 |
| DLK1 | 3 | Mus musculus | ENSMUSG00000040856 | 46 | 90 |
| DLK1 | 3 | Ochotona princeps | ENSOPRG00000012622 | 45 | 89 |
| DLK1 | 3 | Equus caballus | ENSECAG00000012122 | 46 | 90 |
| DLK1 | 2 | Canis familiaris | ENSCAFG00000017925 | 42 | 86 |
| DLK1 | 2 | Felis catus | ENSFCAG00000000458 | 22 | 66 |
| DLK1 | 2 | Ornithorhynchus anatinus | ENSOANG00000005731 | 27 | 71 |
| DLK1 | 3 | Gallus gallus | ENSGALG00000011244 | 51 | 95 |
| DLK1 | 3 | Tetraodon nigroviridis | ENSTNIG00000017282 | 46 | 90 |
| DLK1 | 3 | Takifugu rubripes | ENSTRUG00000009231 | 48 | 92 |
| DLK1 | 3 | Gasterosteus aculeatus | ENSGACG00000009397 | 41 | 85 |
| DLK1 | 4 | Oryzias latipes | ENSORLG00000014546 | 47 | 91 |
| DLK2 | 3 | Homo sapiens | ENSG00000171462 | 49 | 93 |
| DLK2 | 3 | Pan troglodytes | ENSPTRG00000018198 | 49 | 93 |
| DLK2 | 4 | Mus musculus | ENSMUSG00000047428 | 93 | 137 |
| DLK2 | 3 | Canis familiaris | ENSCAFG00000001858 | 49 | 93 |
| DLK2 | 3 | Bos taurus | ENSBTAG00000005850 | 84 | 128 |
| DLK2 | 4 | Gallus gallus | ENSGALG00000010386 | 46 | 90 |
