# Supplementary material for: Exon 6 of human JAG1 encodes a conserved structural unit
Source: BMC Struct Biol. 2009 Jul 8;9:43. doi: 10.1186/1472-6807-9-43 (PMC2725086; doi:10.1186/1472-6807-9-43)
Supplement: Additional file 7 — Sequence alignment. Multiple sequence alignment of the polypeptides encoded by exon 6 of human JAG1 and its homologues in different species. All amino acid sequences annotated in ENSEMBLE as orthologues to JAG1, JAG2, DLL1, DLL4, DLK1, and DLK2 were collected, broken down into segments corresponding to exons, and searched using BLAST with the sequence encoded by exon 6 of human Jagged-1; hits were then aligned using CLUSTAL-W. [file 1472-6807-9-43-S7.doc]

EGF1

EGF2

*JAG1_6 Homo sapiens*  RCQYGWQGLYCDKCIPHPGCVHGICNEPWQCLCETNWGGQLCDKD

*JAG1_6 Macaca mulatta*  RCQYGWQGLYCDKCIPHPGCVHGICNEPWQCLCETNWGGQLCDKD

*JAG1_6 Pan troglodytes*  RCQYGWQGLYCDKCIPHPGCVHGICNEPWQCLCETNWGGQLCDKD

*JAG1_6 Pongo pygmaeus*  RCQYGWQGLYCDKCIPHPGCVHGICNEPWQCLCETNWGGQLCDKD

*JAG1_7 Microcebus murinus*  RCQYGWQGLYCDKCIPHPGCVHGTCNEPWQCLCETNWGGQLCDKD

*JAG1_6 Rattus norvegicus*  RCQYGWQGLYCDKCIPHPGCVHGTCNEPWQCLCETNWGGQLCDKD

*JAG1_6 Mus musculus*  RCQYGWQGLYCDKCIPHPGCVHGTCNEPWQCLCETNWGGQLCDKD

*JAG1_10 Ochotona princeps*  RCQYGWQGLYCDKCIPHPGCVHGTCNEPWQCLCETNWGGQLCDKD

*JAG1_8 Oryctolagus cuniculus*  RCQYGWQGLYCDKCIPHPGCVHGTCNEPWQCLCETNWGGQLCDKD

*JAG1_6 Bos taurus*  RCQYGWQGLYCDKCIPHPGCVHGTCNEPWQCLCETNWGGQLCDKD

*JAG1_5 Equus caballus*  RCQYGWQGLYCDKCIPHPGCVHGTCNEPWQCLCETNWGGQLCDKD

*JAG1_7 Loxodonta africana*  RCQYGWQGLYCDKCIPHPGCVHGTCVEPWQCLCETNWGGQLCDKD

*JAG1_6 Canis familiaris*  RCQYGWQGLYCDKCIPHPGCVHGTCNEPWQCLCETNWGGQLCDKD

*JAG1_6 Felis catus*  RCQYGWQGLYCDKCIPHPGCVHGTCNEPWQCLCETNWGGQLCDKX

*JAG1_5 Myotis lucifugus*  RCQYGWQGLYCDKCIPHPGCVHGTCNEPWQCLCETNWGGQLCDKD

*JAG1_14 Sorex araneus*  SCQYGWQGLYCDKCIPHPGCVHGTCKEPWQCLCETNWGGQLCDKD

*JAG1_6 Dasypus novemcinctus*  XCQYGWQGPYCDKCIPHPGCVHGTCNEPWQCLCETNWGGQLCDKX

*JAG1_6 Monodelphis domestica*  RCQYGWQGQYCDKCIPHPGCVHGTCIEPWQCLCETNWGGQLCDKD

*JAG1_6 Echinops telfairi*  RCQYGWQGLYCDKCIPHPGCVHGTCNEPWQCLCETNWGGQLCDKD

*JAG1_6 Ornithorhynchus anatinus* RCQYGWQGQYCDKCIPHPGCVHGTCNEPWQCLCETNWGGQLCDKV

*JAG1_5 Gallus gallus*  RCQYGWQGQYCDKCIPHPGCVHGTCIEPWQCLCETNWGGQLCDKD

*JAG1_6 Xenopus tropicalis*  RCQYGWQGQYCDKCIPHPGCVHGTCNEPWQCLCETNWGGQLCDKD

*JAG1_6 Oryzias latipes*  DCLYGWQGQYCDKCIPHPGCVHGTCVEPWQCLCETNWGGHLCDKD

*JAG1_6 Tetraodon nigroviridis*  NCLYGWQGQYCDKCIPHPGCVHGTCVEPWQCLCDTNWGGHLCEKD

*JAG1_4 Danio rerio*  RCLYGWQGEYCDQCIPHPGCVHGTCIEPWQCLCDTNWGGQLCDKD

*JAG1_5 Gasterosteus aculeatus*  RCLYGWKGEYCDQCIPHPGCVHGSCVEPWQCLCDTNYGGQLCDKD

*JAG2_6 Homo sapiens*  RCSYGWQGRFCDECVPYPGCVHGSCVEPWQCNCETNWGGLLCDKD

*JAG2_4 Macaca mulatta*  RCSYGWQGRFCDECVPYPGCVHGSCVEPWQCNCETNWGGLLCDKD

*JAG2_4 Pongo pygmaeus*  RCSYGWQGRFCDECVPYPGCVHGSCVEPWQCNCETNWGGLLCDK-

*JAG2_6 Mus musculus*  RCSYGWQGKFCDECVPYPGCVHGSCVEPWHCDCETNWGGLLCDKD

*JAG2_5 Rattus norvegicus*  RCSYGWQGKFCDECVPYPGCVHGSCVEPWHCDCETNWGGLLCDKD

*JAG2_4 Cavia porcellus*  XCSYGWQGRFCDECVPYPGCVHGSCVEPWHCDCETNWGGLLCDKD

*JAG2_4 Equus caballus*  RCSYGWQGRFCDECVPYPGCVHGSCVEPWQCNCETNWGGLLCNKD

*JAG2_1 Bos taurus*  RCSYGWQGRFCDECVPYPGCVHGSCVEPWQCTCETNWGGLLCNKD

*JAG2_4 Canis familiaris*  KCSYGWQGRFCDECVPYPGCVHGSCVDPWQCNCETNWGGLLCNKD

*JAG2_3 Monodelphis domestica*  RCQYGWQGRFCDECVPYPGCLHGSCTEPWKCNCETNWGGLLCDKD

*JAG2_3 Ornithorhynchus anatinus* KCHYGWQGQFCDECVPYPGCLRGSCSEPWQCNCETNWGGLLCNKD

*JAG2_5 Gallus gallus*  KCHYGWQGQYCDECVRYPGCAHGSCNEPWQCNCETNWGGLLCNKD

*JAG2_6 Gasterosteus aculeatus*  KCNYGWQGQFCDECVLYPGCVHGTCNSKWQCNCERNWGGLLCDKD

*JAG2_7 Takifugu rubripes*  TCSYGWDGQFCDECKLFPGCVHGTCVQPWQCRCERNWGGLLCDKD

*JAG2_1 Tetraodon nigroviridis*  RCSFGWEGQFCDECKLYPGCVHGTCNLPWQCNCEKNWGGLLCNKD

*JAG2_6 Oryzias latipes*  KCKYGWDGLLCDQCLPYPGCFHGTCNEPWECSCEKNWGGLLCDKD

*JAG2_6 Danio rerio*  KCNYGWQGQFCDECLPYPGCLHGTCVMPWQCTCEKNWGGLLCDKD

*DLL1_6 Homo sapiens*  KCRVGWQGRYCDECIRYPGCLHGTCQQPWQCNCQEGWGGLFCNQD

*DLL1_5 Macaca mulatta*  KCRVGWQGRYCDECIRYPGCLHGTCQQPWQCNCQEGWGGLFCNQD

*DLL1_6 Pongo pygmaeus*  KCRVGWQGRYCDECIRYPGCLHGTCQQPWQCNCQEGWGGLFCNQD

*DLL1_6 Pan troglodytes*  KCRVGWQGRYCDECIRYPGCLHGTCQQPWQCNCQEGWGGLFCNQD

*DLL1_6 Rattus norvegicus*  KCRVGWQGRYCDECIRYPGCLHGTCQQPWQCNCQEGWGGLFCNQD

*DLL1_6 Mus musculus*  KCRVGWQGRYCDECIRYPGCLHGTCQQPWQCNCQEGWGGLFCNQD

*DLL1_6 Oryctolagus cuniculus*  XCRVGWQGRYCDECIRYPGCLHGTCQQPWQCNCQEGWGGLFCNQD

*DLL1_6 Bos taurus*  KCRVGWQGRYCDQCIRYPGCLHGTCRQPWQCNCQEGWGGLFCNQD

*DLL1_6 Canis familiaris*  KCRVGWQSRYCDQCIRYPGCLHGTCQQPWQCNCQEGWGGLFCNQD

*DLL1_3 Felis catus*  KCRVGWQSRYCDQCIRYPGCLHGTCQQPWQCNCQEGWGGLFCNQD

*DLL1_7 Sorex araneus*  XCRVGWQGRYCDQCIRYPGCLHGTCQQPWQCNCQEGWGGLFCNQD

*DLL1_6 Monodelphis domestica*  KCRVGWQGRYCDECIRYPGCLHGTCQQPWQCNCQEGWGGLFCNQD

*DLL1_2 Erinaceus europaeus*  KCRVGWQGRYCDQCIRYPGCLHGTCQQPWQCTCQEGWGGLFCNQD

*DLL1_4 Myotis lucifugus*  KCRVGWQGRYCDQCIRYPGCLHGTCQQPWQCTCQEGWGGLFCNQD

*DLL1_6 Gallus gallus*  KCRVGWQGRYCDECIRYPGCLHGTCQQPWQCNCQEGWGGLFCNQD

*DLL1_6 Xenopus tropicalis*  KCRVGWQGRYCDECIRYPGCLHGTCQQPWQCNCQEGWGGLFCNQD

*DLL1_5 Tupaia belangeri*  XCRVGWQGRYCDECIRYPGCLHGTCQQPWQCNCQEGWGGLFCNQD

*DLL1_6 Takifugu rubripes*  KCRVGFKGRYCDECIRYPGCLHGTCQQPWQCNCQEGWGGLFCNQD

*DLL1_6 Oryzias latipes*  KCRVGFSGRYCDDCIRYPGCLHGTCQQPWQCNCQEGWGGLFCNQD

*DLL1_6 Gasterosteus aculeatus*  KCRVGFSGRYCDDCIRYPGCLHGTCQQPWQCNCQEGWGGLFCNQD

*DLL4_6 Homo sapiens*  LCRPGWQGRLCNECIPHNGCRHGTCSTPWQCTCDEGWGGLFCDQD

*DLL4_7 Pan troglodytes*  LCRPGWQGRLCNECIPHNGCRHGTCSTPWQCTCDEGWGGLFCDQD

*DLL4_6 Macaca mulatta*  LCRPGWQGRLCNECIPHNGCRHGTCSTPWQCTCDEGWGGLFCDQD

*DLL4_7 Otolemur garnettii*  LCRPGWQGRLCNECIPHNGCRHGTCSTPWQCTCDEGWGGLFCDQD

*DLL4_6 Microcebus murinus*  LCRPGWQGRLCNKCIPHNGCRHGTCSSPWQCTCDEGWGGLFCDQD

*DLL4_6 Rattus norvegicus*  NCRPGWQGPLCNECIPHNGCRHGTCTIPWQCACDEGWGGLFCDQD

*DLL4_6 Mus musculus*  ICRPGWQGRLCNECIPHNGCRHGTCSIPWQCACDEGWGGLFCDQD

*DLL4_4 Cavia porcellus*  LCRPGWQGRLCNECIPHNGCRHGTCSTPWQCTCNEGWGGLFCDQD

*DLL4_11 Ochotona princeps*  LCRPGWQGRLCNECIPHNGCRHGTCSIPWQCTCDEGWGGLFCDQD

*DLL4_7 Oryctolagus cuniculus*  LCRPGWQGRLCNECIPHNGCRHGTCSIPWQCTCDEGWGGLFCDQD

*DLL4_6 Equus caballus*  ICRPGWQGRLCNECIPHNGCRHGTCNTPWQCTCDEGWGGLFCDQD

*DLL4_6 Bos taurus*  ICRPGWQGRLCNECIPHNGCRHGTCSTPWQCTCDEGWGGLFCDQD

*DLL4_6 Canis familiaris*  ICRPGWQGRLCNECIPHNGCRHGTCSIPWQCTCDEGWGGLFCDQD

*DLL4_9 Felis catus*  ICRPGWQGRLCNECIPHNGCRHGTCSIPWQCTCDEGWGGLFCDQX

*DLL4_3 Myotis lucifugus*  ICRPGWQGRLCNECIPHNGCRHGTCTTPWQCTCDEGWGGLFCDQD

*DLL4_6 Sorex araneus*  ICRPGWQGRLCNECIPHNGCRHGTCSIPWECNCDEGWGGLFCDQD

*DLL4_6 Monodelphis domestica*  LCRPGWQGRLCDKCIPHNGCRHGTCSIPWQCTCNEGWGGLFCDQD

*DLL4_3 Erinaceus europaeus*  SCRPGWQGPLCNECIPHNGCHHGTCSIPWECNCDEGWGGLFCDQD

*DLL4_16 Tupaia belangeri*  LCRPGWQGRLCNECIPHNGCRHGTCSTPWQCTCDEGWGGLFCDQD

*DLL4_3 Ornithorhynchus anatinus* LCRPGWQGRLCDRCIPHVGCRHGTCSIPWQCTCDEGWGGLFCDQD

*DLL4_6 Gallus gallus*  ICRSGWQGRYCDECIPHIGCRHGTCKTQWQCICDEGWGGLFCDQD

*DLL4_5 Xenopus tropicalis*  TCRPGWQGRFCNECIPHNGCRHGTCQIQWQCICDEGWGGLFCDQD

*DLL4_5 Gasterosteus aculeatus*  VCREGWQGTFCDECKKYPACKHGTCQLPWQCNCQEGWGGLLCDQD

*DLL4_6 Danio rerio*  VCREGWQGKFCTECKTYPACKHGTCHLPGQCNCKEGWGGLFCDQD

*DLL4_8 Oryzias latipes*  VCRKGWTGMFCDECETYPACNHGTCQLPWQCNCQEGWGGLLCDQD

*DLL4_8 Takifugu rubripes*  KCREGWQGLFCDVCKLHPSCKHGTCNEPWQCICKEGWGGIYCNQD

*DLL4_6 Tetraodon nigroviridis*  KCRKGWQGPSCDVCEVHPSCKHGTCNEPWQCICKQGWGGIYCNQD

*DLK1_3 Homo sapiens*  RCQPGWQGPLCDQCVTSPGCLHGLCGEPGQCICTDGWDGELCDRD

*DLK1_3 Macaca mulatta*  RCQPGWQGPLCDQCVTSPGCLHGLCEEPWQCICTDGWDGKLCDRD

*DLK1_3 Pongo pygmaeus*  RCQPGWQGPLCDQCMTSPGCLHGLCEEPGQCICTDGWDGELCDRD

*DLK1_1 Otolemur garnettii*  -CQPGWQGPLCDQCMTAPGCYNGYCEEAWQCICNDGWEGKLCEID

*DLK1_3 Rattus norvegicus*  RCEPGWEGPLCEKCVTSPGCVNGLCEEPWQCVCKEGWDGKFCEID

*DLK1_3 Mus musculus*  RCHVGWEGPLCDKCVTAPGCVNGVCKEPWQCICKDGWDGKFCEID

*DLK1_3 Ochotona princeps*  RCQPGWQGPLCDQCVTSPGCMNGFCEEPWQCLCQEGWDGKLCDTD

*DLK1_3 Equus caballus*  RCQPGWQGPLCDQCVTFPGCVHGLCVEPWQCICDDGWDGNLCDLD

*DLK1_2 Canis familiaris*  RCQPGWQGPLCDQCVTFPGCVNGLCVEPWQCICDDGWDGNLCDID

*DLK1_2 Felis catus*  RCQPGWQGPLCEQCVTFPGCVNGLCVEPWQCVCDDGWNGKLCDID

*DLK1_2 Ornithorhynchus anatinus*  RCQPGWRGPLCTECIPFPGCLHGGCTLPWQCVCQEGWVGSLCDID

*DLK1_3 Gallus gallus*  RCLPGWQGALCNQCVPFPGCLHGSCVKPWQCICEEGWVGSLCDID

*DLK1_3 Tetraodon nigroviridis*  RCKPGWQGENCDQCVPFPGCLHGKCEKAWQCVCEEGWVGSLCDQD

*DLK1_3 Takifugu rubripes*  RCKPGWQGENCDQCVPFPGCLHGKCEKAWQCVCEEGWVGSLCDQD

*DLK1_3 Gasterosteus aculeatus*  RCKPGWQGFNCEQCVRFPGCLHGACEKAWQCVCEDGWAGSLCDQD

*DLK1_4 Oryzias latipes*  RCKPGWEGENCDRCIPFPGCLHGSCEKAWQCICKEGWVGSLCDQD

*DLK2_3 Homo sapiens*  RCDPGWEGLHCERCVRMPGCQHGTCHQPWQCICHSGWAGKFCDKD

*DLK2_3 Pan troglodytes*  RCDPGWEGLHCERCVRMPGCQHGTCHQPWQCICHSGWAGKFCDKD

*DLK2_4 Mus musculus*  RCDPGWEGLHCERCVRMPGCQHGTCHQPWQCICHSGWAGKFCDKD

*DLK2_3 Canis familiaris*  RCDPGWEGLHCERCVRMPGCQHGTCHQPWQCICHSGWAGKFCDKD

*DLK2_3 Bos taurus*  RCDPGWEGLHCERCVRMPGCQHGTCHQPWQCICHTGWAGKFCDKD

*DLK2_4 Gallus gallus*  RCDPGWEGDYCEECVRMPGCLHGTCHQPWQCICHSGWAGKFCDKD
