# Supplementary material for: Exon 6 of human JAG1 encodes a conserved structural unit
Source: BMC Struct Biol. 2009 Jul 8;9:43. doi: 10.1186/1472-6807-9-43 (PMC2725086; doi:10.1186/1472-6807-9-43)
Supplement: Additional file 8 — Sequence comparison. Multiple sequence alignment of the amino acid sequence encoded by exon 6 in JAG1 and its homologues compared to the sequence of the DSL domain; the disulfide topology in the DSL and J1ex6 region of human Jagged-1 is also shown. [file 1472-6807-9-43-S8.pdf]

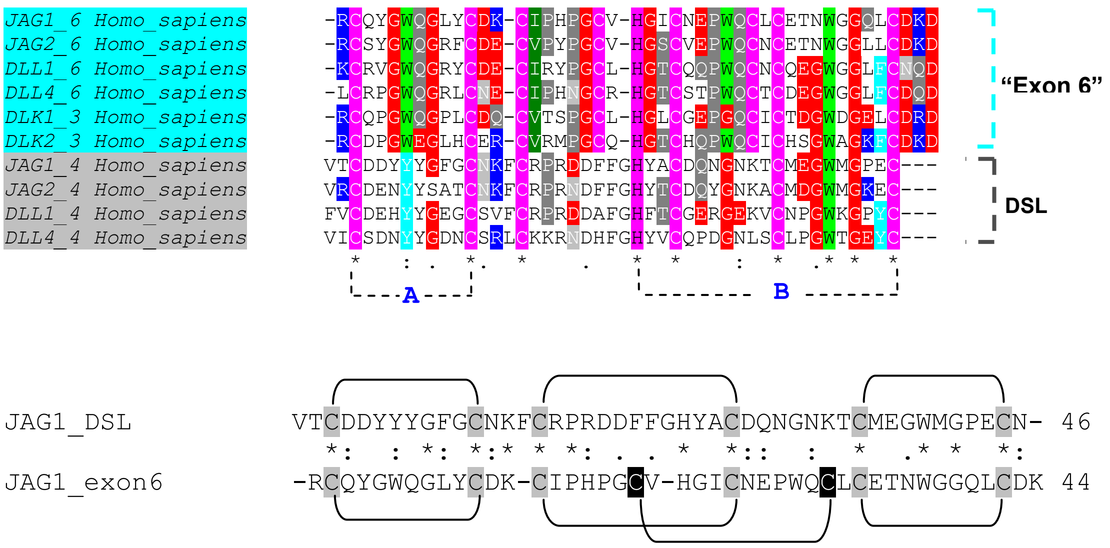
JAG1_6 Homo_sapiens -RCQYGWQGLYCDK-CIPHPGCV-HGICNEPWQCLCETNWGGQLCDKD
JAG2_6 Homo_sapiens -RCSYGWQGRFCDE-CVPYPGCV-HGSCVEPWQCNCETNWGGLLCDKD
DLL1_6 Homo_sapiens -KCRVGWQGRYCDE-CIRYPGCL-HGTCQQPWQCNCQEGWGGLFCNQD
DLL4_6 Homo_sapiens -LCRPGWQGRLCNE-CIPHNGCR-HGTCSTPWQCTCDEGWGGLFCDQD
DLK1_3 Homo_sapiens -RCQPGWQGPLCDQ-CVTSPGCL-HGLCGEPGQCICTDGWDGELCDRD
DLK2_3 Homo_sapiens -RCDPGWEGLHCER-CVRMPGCQ-HGTCHQPWQCICHSGWAGKFCDKD
JAG1_4 Homo_sapiens VTCDDYYYGFGCNKFCRPRDDFFGHYACDQNGNKTCMEGWMGPEC---
JAG2_4 Homo_sapiens VRCDENYYSATCNKFCRPRNDFFGHYTCDQYGNKACMDGWMGKEC---
DLL1_4 Homo_sapiens FVCDEHYYGEGCSVFCRPRDDAFGHFTCGERGEKVCNPGWKGPYC---
DLL4_4 Homo_sapiens VICSDNYYGDNCSRLCKKRNDHFGHYVCQPDGNLSCLPGWTGEYC---
“Exon 6”
DSL
A
B
JAG1_DSL VTCDDYYYGFGCNKFCRPRDDFFGHYACDQNGNKTCMEGWMGPECN- 46
JAG1_exon6 -RCQYGWQGLYCDK-CIPHPGCV-HGICNEPWQCLCETNWGGQLCDK 44
